# Association between oxidative stress and liver fibrosis severity in non-alcoholic fatty liver disease: insights from the pro-oxidant antioxidant balance method in a population from Tehran and Mashhad, Iran

**DOI:** 10.3389/fmed.2025.1539605

**Published:** 2025-03-12

**Authors:** Hami Ashraf, Amir Anushiravani, Maryam Rayatpisheh, Daryoush Hamidi Alamdari, Arianaz Hossieni, Behrang Kazeminezhad

**Affiliations:** ^1^Chronic Respiratory Diseases Research Center, National Research Institute of Tuberculosis and Lung Diseases (NRITLD), Shahid Beheshti University of Medical Sciences, Tehran, Iran; ^2^Innovative Laboratory Assays in Biomedicine Research Center, Shahid Beheshti University of Medical Sciences, Tehran, Iran; ^3^Digestive Diseases Research Institute, Tehran University of Medical Science, Tehran, Iran; ^4^Surgical Oncology Research Center, Mashhad University of Medical Sciences, Mashhad, Iran; ^5^Modarres Hospital, School of Medicine, Shahid Beheshti University of Medical Sciences, Tehran, Iran

**Keywords:** non-alcoholic fatty liver, liver fibrosis, pro-oxidant-antioxidant balance, cross-sectional study, non-alcoholic fatty liver disease (NAFLD)

## Abstract

**Background:**

The exact mechanisms of non-alcoholic fatty liver disease (NAFLD), recently redefined as metabolic dysfunction-associated steatotic liver disease (MASLD), remain unclear. However, oxidative stress is recognized as a factor across all stages of NAFLD. The Pro-oxidant Antioxidant Balance (PAB) method is an important clinical tool that provides an assessment of the balance between oxidants and antioxidant. We aimed to explore oxidative stress in NAFLD using the PAB method.

**Methods:**

Individuals with NAFLD were recruited in 2021. Eligible participants underwent detailed assessments, including liver elastography for fibrosis evaluation. Blood samples (5 mL) were collected to measure serum PAB levels. The METAVIR score, derived from FibroScan measurements of liver stiffness, categorized fibrosis severity from F0 (no fibrosis) to F4 (advanced fibrosis or cirrhosis).

**Results:**

The study included 102 participants, with a mean age of 50.12 ± 10.03 years. Significant correlations were observed between FibroScan scores and variables such as age, body mass index (BMI), history of chronic diseases, and family history of NAFLD. PAB levels were notably higher in patients with advanced fibrosis (F2 and F3 groups: 86.32 ± 25.53) compared to those in early stages (F0 and F1 groups: 45.36 ± 21.29). Moreover, FibroScan scores showed a significant positive association with PAB values (odds ratio [OR]: 1.07; 95% confidence interval (CI): 1.04, 1.10), even after adjusting for confounding variables (OR: 1.13; 95% CI: 1.07, 1.18).

**Conclusion:**

Elevated PAB levels were strongly associated with advanced stages of liver fibrosis in NAFLD patients, reflecting increased oxidative stress with disease progression. These results highlight the potential of PAB as a marker for monitoring oxidative stress and disease severity in NAFLD. Nevertheless, further large-scale studies are warranted.

## Introduction

Non-alcoholic fatty liver disease (NAFLD), recently redefined as metabolic dysfunction-associated steatotic liver disease (MASLD), has a spectrum of liver disorders, which ranges from benign hepatic steatosis to non-alcoholic steatohepatitis (NASH), fibrosis, cirrhosis, and hepatocellular carcinoma (1). It is a progressive and multifactorial condition associated with metabolic syndrome, with main contributors including insulin resistance, mitochondrial dysfunction, and oxidative stress (1). NAFLD is estimated to have an incidence of 47 cases per 1,000 people, with higher rates in males (2). According to a meta-analysis from 2000 to 2022, the overall prevalence was about 33% in Iran (3).

The pathogenesis of NAFLD is multi-factorial, with “two-hit hypothesis” describing lipid accumulation as the initial “hit,” which sensitizes hepatocytes to subsequent injuries such as oxidative stress and inflammation (4). A refined “multi-hit hypothesis” provides mechanisms including altered adipocytokine levels, mitochondrial dysfunction, and endoplasmic reticulum stress in disease progression (5). Oxidative stress (OS) plays a central role in advancing steatosis to NASH, triggering lipid peroxidation, inflammation, and fibrogenesis via reactive oxygen species (ROS) and imbalances in antioxidant defenses (6). Studies have shown that oxidative stress not only contributes to the development of NAFLD but also regulates inflammatory pathways, particularly through transcription factors such as NFκB, which promote disease progression (7).

Advancing age, central obesity, and components of metabolic syndrome, such as dyslipidemia and hyperglycemia, are risk factors for NAFLD and fibrosis progression (8). The severity of fibrosis is the primary determinant of prognosis, with advanced fibrosis associated with higher mortality (9). Despite its global prevalence and clinical impact, NAFLD remains underdiagnosed, and there is no widely accepted pharmacological treatment. Therefore, lifestyle modifications remain the main management (10).

The role of oxidative stress in NAFLD progression highlights the need for reliable, cost-effective methods to evaluate the effects. Conventional methods to measure OS have focused on isolated markers of oxidative damage or antioxidant defenses, which limits their application in assessing the net oxidative balance (11). The Pro-oxidant Antioxidant Balance (PAB) test, which simultaneously measures the equilibrium between oxidants and antioxidants in a single assay, provides a novel approach to overcome this limitation. Unlike other oxidative stress markers that assess individual components of the redox system, the PAB method provides a more comprehensive measure of systemic oxidative stress, making it particularly useful in conditions where oxidative imbalance plays a pivotal role, such as NAFLD (12). In addition, our study provides several novel aspects that differentiate it from existing literature. It is one of the few studies in Iran to specifically focus on the relationship between oxidative stress and liver fibrosis severity in NAFLD patients, using the PAB method as the primary tool. Furthermore, while many studies have assessed oxidative stress in liver diseases, few have linked this to the non-invasive FibroScan results for accurate staging of fibrosis. This integration of PAB with FibroScan scores provides a more nuanced understanding of how oxidative stress correlates with fibrosis severity in NAFLD, particularly in an Iranian cohort. The inclusion of participants from Tehran and Mashhad enhances the generalizability of the results within the Iranian population, making our study more regionally relevant and adding to the existing body of research.

This study aimed to explore oxidative stress in NAFLD using the PAB method, with a focus on its relationship to fibrosis scores. By examining correlations with age, sex, body mass index (BMI), and clinical history, the study aimed to determine the contribution of oxidative stress to disease severity and identify potential avenues for clinical assessment and management.

## Methods

### Study participants

This cross-sectional study was conducted to compare oxidative stress levels in patients with NAFLD and healthy individuals. The study enrolled 104 participants from hepatology clinics at Shariati Hospital in Tehran and Imam Reza Hospital in Mashhad, Iran, in 2021. All participants were adults (≥18 years) and had undergone ultrasound elastography for the diagnosis of NAFLD. Patients with other severe liver diseases (e.g., viral hepatitis, autoimmune hepatitis, cirrhosis), kidney diseases (e.g., chronic kidney disease, nephrotic syndrome), metabolic disorders (e.g., uncontrolled diabetes mellitus), hematological diseases (e.g., anemia, leukemias, coagulopathies), or malignant disorders were excluded. Additionally, participants with significant alcohol consumption, history of hepatotoxic drug use, pregnancy, or systemic inflammatory conditions were excluded to minimize confounding factors related to oxidative stress. Only participants with confirmed NAFLD were included, and those with any co-existing liver disease or severe systemic conditions were excluded to ensure a clear diagnosis of NAFLD.

Based on ultrasound elastography results, patients were categorized into three groups: normal population and early stages of fatty liver (FibroScan score 0 or 1), fatty liver with moderate fibrosis (FibroScan score 2), and fatty liver with severe fibrosis (FibroScan score 3). Fibrosis severity was further defined according to the METAVIR scoring system, with stages F0 to F1 representing no fibrosis or mild fibrosis, F2 indicating moderate fibrosis, and F3 corresponding to severe fibrosis. Data on age, sex, medical history, and family medical history were collected from medical records.

This study received approval from the Ethics Committee of Shahid Beheshti University of Medical Sciences (IR.SBMU.MSP.REC.1400.064). All procedures followed relevant guidelines and regulations. Informed consent was obtained from all participants.

### Sample preparation and collection

We used high-grade chemicals, including tetramethylbenzidine (TMB) powder (3,3′,5,5′-Tetramethylbenzidine, Fluka), peroxidase enzyme (Applichem: 230 U/mg, A3791,0005, Darmstadt, Germany), chloramine T trihydrate (Applichem: A4331, Darmstadt, Germany), and hydrogen peroxide (30%; Merck). All other reagents were of analytical grade and prepared using double-distilled water.

For sample collection, 5 mL of blood was received from each participant following an overnight fast to conduct the PAB assay. The samples were immediately centrifuged at 2,000 × g for 15 min, after which serum aliquots were separated and stored at −70°C. To evaluate inter-assay precision, three aliquots were prepared from each set of 10 samples.

### Measuring PAB levels

Serum PAB levels were determined following the method described by Alamdari et al. (13). This assay uses TMB and its cation as a redox indicator involved in two rapid reactions. Standard solutions were prepared by mixing various ratios of 1 mM hydrogen peroxide with 6 mM uric acid in 10 mM NaOH. A TMB solution was prepared by dissolving a TMB tablet in 10 ml of substrate buffer (0.05 mmol citrate phosphate buffer, pH = 5). TMB cations were generated by adding 18 microliters of fresh chloramine solution to 1 mL of TMB solution and incubating for 20 min. Subsequently, 1.25 units of peroxidase enzyme solution were added to 9 mL of TMB solution. The working solution was created by combining these two solutions, incubating for 5 min at room temperature. A mixture of 10 microliters of sample, standard, or blank (distilled water) and 200 μL of working solution was added to each well of a 96-well plate. The plate was incubated in the dark at room temperature for 12 min. Following incubation, 100 μL of 2N HCl was added to each well, and the plate was left in the dark for an additional 45 min. Absorbance was measured at 450 nm using an ELISA reader, with a reference wavelength of 620 or 570 nm. Sample values were interpolated from a standard curve generated using the standard solutions. PAB values were expressed in arbitrary HK units, calculated as the percentage of hydrogen peroxide in the standard solution multiplied by six. Unknown sample values were determined based on the standard curve.

### Determining METAVIR score

The METAVIR score was derived from FibroScan output, a specialized ultrasound device for assessing liver heath by measuring fibrosis and steatosis. Fibrosis results were expressed in kilopascals (kPa), which ranged from 2 to 6 kPa under normal conditions, with a maximum measurable value of 75 kPa. Elevated kPa values indicate liver disease. The METAVIR scoring system quantifies fibrosis severity on a scale from F0 to F4, based on the measured kPa values: F0 to F1 corresponds to 2–7 kPa, indicating no fibrosis or mild fibrosis; F2 corresponds to 7.5–10 kPa, signifying moderate fibrosis; F3 corresponds to 10–14 kPa, representing severe fibrosis; and F4 corresponds to values ≥14 kPa, showing advanced fibrosis or cirrhosis.

### Statistical analysis

The study included 104 patients, of whom 102 had valid FibroScan measurements. Although the sample size may be considered modest, it was determined based on power calculations to detect meaningful differences in PAB levels across fibrosis stages. In addition, our study reflects real-world clinical practice, and subgroup analyses were conducted with appropriate statistical corrections to minimize bias. Data analysis was conducted using SPSS software, version 16 (SPSS Inc., Chicago, IL, USA). Quantitative variables were expressed as mean ± standard deviation (SD), while qualitative variables were reported as frequency and percentage. The distribution of variables was assessed using the Kolmogorov-Smirnov test. To compare proportions and means between the experimental and control groups, the Chi-squared test was employed for qualitative variables, and the independent *t*-test was applied for quantitative variables. Moreover, multiple linear regression models were used to examine the associations between serum PAB levels and liver fibrosis, adjusting for potential confounders, including age, sex, BMI, and comorbidities. To assess multicollinearity, variance inflation factors were calculated for the independent variables, and none exceeded the threshold of 10, indicating no significant multicollinearity among the variables.

## Results

### Participant characteristics and fibrosis distribution

Out of the 104 patients enrolled in the study, 102 were successfully evaluated for liver fibrosis using FibroScan. The participants were mostly men (*n* = 75), with an overall mean age of 50.6 years. Among these patients, 65 individuals (63.7%) were categorized with a METAVIR fibrosis score of F0 or F1, as the control group. Another 27 individuals (26.5%) had a score of F2, while 10 patients (9.8%) were in the F3 group. No patients were classified with a score of F4 (Table 1).

**Table 1:** General characteristics of the study population (*n* = 102) and analysis of demographic, anthropometric, and serologic parameters across FibroScan score.

		**Total**	**FibroScan score 0, 1**	**FibroScan R score 2**	**FibroScan score 3**	***P*** **-value**	
						***P***	***P***
Age (year)		50.12 ± 10.03	52.41 ± 9.62	45.37 ± 7.97	48.10 ± 13.30	0.005^*^	0.004^#^
Sex	Male	75 (73.5)	52 (80)	19 (70.4)	4 (40)	0.043^**^	0.053^&^
	Female	27 (26.5)	13 (20)	8 (29.6)	6 (60)		
History of chronic diseases	No	61 (59.8)	45 (69.2)	16 (43.2)		0.011^**^	–
	Thyroid disorder	7 (6.9)	2 (3.1)	5 (13.5)			
	HTN	14 (13.7)	7 (10.8)	7 (18.9)			
	DM	6 (5.9)	3 (4.6)	3 (8.1)			
	CAD	4 (3.9)	2 (3.1)	2 (5.4)			
	PUD	4 (3.9)	3 (4.6)	1 (2.7)			
	Others	6 (5.9)	3 (4.6)	3 (8.1)			
Family history of fatty liver	Negative	76 (74.5)	54 (83.1)	16 (59.3)	6 (60.0)	0.011^**^	0.010^&^
	Positive	26 (25.5)	11 (16.9)	11 (40.7)	4 (40.0)		
BMI		29.63 ± 4.70	28.13 ± 3.62	31.29 ± 4.54	35.00 ± 6.35	0.001^*^	< 0.001^#^
PAB		60.22 ± 30.18	45.36 ± 21.29	78.31 ± 21.59	107.95 ± 23.39	0.001^*^	0.001^#^

BMI, Body Mass Index; PAB, Pro-oxidant Antioxidant Balance; HTN, Hypertension; DM, Diabetes Mellitus; PUD, Peptide Ulcer Disease; CAD, Coronary Artery Diseases.

^*^P-value was reported based on ANOVA test; ^**^P-value was reported based on Chi-square test; ^#^*P*-value was reported based on ANCOVA after adjustment of age and sex; ^&^*P*-value was reported based on binary regression.

The quantitative variables are presented as mean ± standard deviation. The qualitative variables are presented as frequency (percent). Groups were defined according to the FibroScan scores, with fibrosis severity classified as follows: F0 (no fibrosis), F1 (mild fibrosis), F2 (moderate fibrosis), and F3 (severe fibrosis).

Sixty-one patients (59.8%) had no history of chronic diseases. However, certain conditions, including hypo- or hyperthyroidism (*n* = 7), hypertension (*n* = 14), diabetes (*n* = 6), coronary artery disease (*n* = 4), and peptic ulcer disease (*n* = 4) were observed. Family history of NAFLD was reported by 26 patients (25.5%). Among the 39 patients with METAVIR scores of F2 or F3, a greater prevalence of chronic diseases and a family history of NAFLD was noted compared to those in the F0 and F1 categories (*P* < 0.05; Table 1).

### Fibrosis stage and sex analysis

In the two-group analysis (F0 and F1 vs. F2 and F3), women accounted for 20% of the F0 and F1 group but represented 37.8% of the F2 and F3 group, a statistically significant difference (*P* = 0.043). Similarly, in the three-group analysis, women comprised 30% of the F2 group and 60% of the F3 group (Table 1).

### Age and fibrosis progression

A significant difference in mean age was observed between fibrosis groups. The mean age in the F0 and F1 group was 52.41 ± 9.62 years, compared to 46.10 ± 9.57 years in the F2 and F3 group (*P* = 0.002). After adjusting for sex, the mean ages were 52.35 ± 1.35 years and 46.49 ± 1.59 years for the F0 and F1 and F2 and F3 groups, respectively (*P* = 0.001). The three-group analysis showed the F2 group had a significant lower mean age (45.37 ± 7.97 years) than the F0 and F1 (52.41 ± 9.62 years) and F3 groups (48.10 ± 13.30 years; Table 1).

### Comorbidities and fibrosis stage

A higher prevalence of chronic diseases was shown in people with more advanced fibrosis. In the two-group analysis, 30.8% of individuals in the F0 and F1 group had a history of chronic illness compared to 56.8% in the F2 and F3 group (*P* = 0.009). By disease, the prevalence of hypertension, thyroid disorders, and diabetes was higher in the F2 and F3 groups. Notably, among the F3 patients, 20% reported thyroid disorders and coronary artery disease, with 40% having no comorbidities (Table 1).

### Body mass index and fibrosis progression

BMI increased significantly with fibrosis severity. The mean BMI in the F0 and F1 group was 28.13 ± 3.62 kg/m^2^ compared to 32.28 ± 5.25 kg/m^2^ in the F2 and F3 group (*P* < 0.001). The three-group analysis showed higher BMI values in the F0 and F1, F2, and F3 groups, at 28.13 ± 3.62, 31.29 ± 4.54, and 35.00 ± 6.35 kg/m^2^, respectively, with significant differences between all three groups (*P* < 0.001; Table 1).

### Serum PAB levels and fibrosis

**Figure 1:**
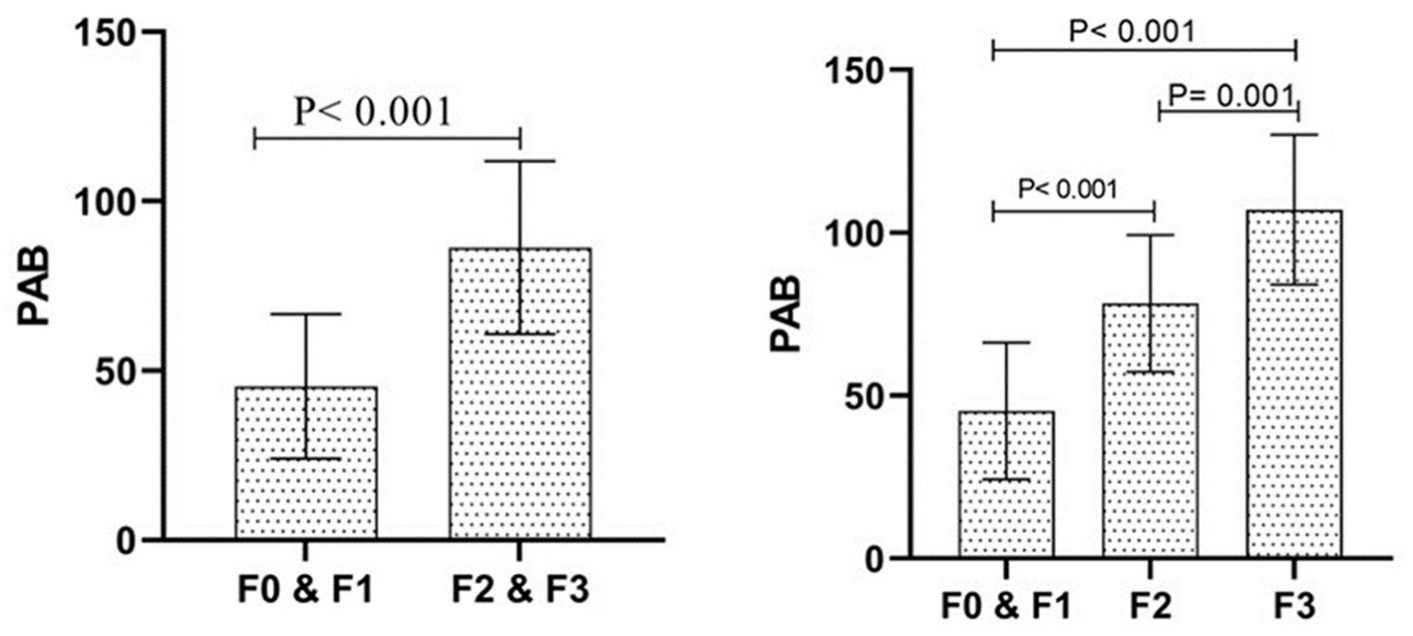
The status of Pro-oxidant Antioxidant Balance (PAB) between the investigated groups. The description of the data has been done using the mean and standard deviation. Data analysis was done using the Independent-Sample *T*-test. Groups were defined according to the FibroScan scores, with fibrosis severity classified as follows: F0 (no fibrosis), F1 (mild fibrosis), F2 (moderate fibrosis), and F3 (severe fibrosis).

The PAB levels were significantly higher in individuals with advanced fibrosis. The mean serum PAB in the F0 and F1 group was 45.36 ± 21.29, compared to 86.32 ± 25.53 in the F2 and F3 group (*P* < 0.001). The three-group analysis revealed mean PAB levels of 45.36 ± 21.29, 78.31 ± 21.59, and 107.95 ± 23.39 in the F0 and F1, F2, and F3 groups, respectively, with significant differences among all three groups (*P* < 0.001; Figure 1).

Importantly, after adjusting for potential confounders, including age, sex, BMI, and family history, PAB levels remained significantly associated with fibrosis severity, with adjusted mean values of 57.99 ± 2.41 in the F0 and F1 group, 82.72 ± 3.31 in the F2 group, and 104.25 ± 5.05 in the F3 group. These findings suggest that PAB may serve as a potential biomarker for fibrosis progression (Figure 1).

### Predictors of fibrosis risk and diagnostic potential of PAB

Regression analysis revealed that while sex did not predict fibrosis risk (*P* > 0.05), age was inversely associated with fibrosis, with a 7% reduction in risk per additional year of age [odds ratio [OR]: 0.93; 95% confidence interval (CI): 0.88, 0.97]. PAB was initially found to be an independent predictor, with a 7% increased risk of fibrosis per unit increase in PAB (OR: 1.07; 95% CI: 1.04, 1.10). However, after adjusting for potential confounders—such as age, sex, BMI, family history of NAFLD, and other comorbidities—the association between PAB and fibrosis risk was strengthened, with a 13% increased risk per unit increase in PAB (OR: 1.13; 95% CI: 1.07, 1.18). These results suggest that the relationship between PAB and fibrosis severity is robust and remains significant even after accounting for common confounding factors. Moreover, multicollinearity diagnostics showed that no variable had a value >10, suggesting that multicollinearity did not significantly affect the regression models (Table 2).

**Table 2:** Analysis of the association between various factors and the risk of liver fibrosis.

**Variable**	**OR (95% CI)**	***P*-value**
**Sex**		
**Male (reference)**	**Ref**	**Ref**
Female	2.43 (0.98, 5.99)	0.053
**Family history of fatty liver**		
**No (reference)**	**Ref**	**Ref**
Yes	3.34 (1.33, 8.42)	0.010
Age	0.93 (0.88, 0.97)	0.004
PAB	1.07 (1.04, 1.10)	< 0.001
Adjusted PAB	1.13 (1.07, 1.18)	< 0.001

OR, odds ratio; CI, confidence interval; PAB, Pro-oxidant Antioxidant Balance.

## Discussion

This study aimed to investigate the association between serum PAB levels and liver fibrosis status. The findings revealed that higher serum PAB levels and family history of NAFLD were independently associated with the progression of liver fibrosis. Additionally, there was a marginally non-significant association between being female and an increased risk of fibrosis. Conversely, an inverse relationship was observed between age and the risk of liver fibrosis.

In this study, a non-significant association was found between female sex and an increased risk of liver fibrosis, but it was near to significance. Comparing this result with previous studies, the evidence on the association between sex and liver fibrosis is mixed. A systematic review and meta-analysis found that women have a 19% lower likelihood of developing NAFLD compared to men [risk ratio (RR): 0.81; 95% CI: 0.68–0.97; 14]. However, once NAFLD is present, their risk of progressing to advanced fibrosis is 37% higher than that of men (RR: 1.37; 95% CI: 1.12–1.68), with this disparity becoming more pronounced after the age of 50 years (14). These findings suggest that hormonal and metabolic changes, particularly the decline in estrogen during menopause, may play a critical role in liver disease progression among women. Accordingly, NAFLD is influenced by complex interactions of metabolic and hormonal factors, with estrogen having protective effects against NAFLD and its progression in premenopausal women (15). However, estrogen deficiency post-menopause accelerates metabolic dysfunction and fibrotic liver changes (15). The variabilities may be due to differences in study designs, population characteristics, and the role of metabolic factors. While research on NAFLD has made significant progress, there remains a gap in understanding sex differences in NAFLD risk factors. Most clinical and epidemiological studies did not address these differences, particularly the role of sex hormones, menopausal status, and age (16). Future studies should focus on sex-specific mechanisms and incorporate these factors into clinical trials and gene association studies to improve therapeutic outcomes and guide more personalized treatments for both men and women (16).

Our study found that individuals with normal fibrosis (F0 and F1) were significantly older compared to those with liver fibrosis (F2 and F3). Even when stratifying the fibrotic group into F2 and F3 subgroups, the average age of these groups remained lower than the control group. This finding contrasts with general trends in the literature, where age is typically recognized as a risk factor for the development of liver fibrosis, especially in NAFLD. In this regard, a meta-analysis revealed that age was significantly associated with the presence of NAFLD, as older individuals showed higher levels of metabolic disturbances (17). Age-related changes, such as decreased liver function, reduced hepatic blood flow, and alterations in lipid metabolism, are often observed with advancing age, making older individuals more susceptible to liver damage (18). However, the relatively younger age of fibrotic patients in this study may be attributed to the small sample size of the fibrosis groups, which limits the generalizability of the result.

Our study determined family history of NAFLD as an associate of NAFLD in the included population. It can be explained by the roles of genetic factors in the development and progression of NAFLD. Genetic factors could play a crucial role in the development of liver fibrosis in NAFLD, particularly in Iran, where hereditary components may contribute to an earlier onset of fibrosis despite younger age (19). The genetic predisposition to NAFLD and its progression has been supported by studies indicating strong familial associations, particularly in cases of children with overweight or lipid metabolism disorders (20, 21).

Our study found a positive association between PAB levels and fibrosis scores in patients with NAFLD. Specifically, serum PAB levels were significantly higher in the F3 group compared to both the F2 group and the combined F0 and F1 groups, even after adjusting for confounding variables. The observed elevation in PAB levels among individuals with advanced fibrosis aligns with previous research highlighting the role of oxidative stress in liver disease progression. Oxidative stress has been implicated in hepatocellular damage, inflammation, and fibrosis through mechanisms involving lipid peroxidation, protein oxidation, and mitochondrial dysfunction. The PAB assay provides an integrative measure of the balance between oxidants and antioxidants, reflecting systemic oxidative stress status. In this regard, Franceschetti et al. highlighted the critical role of oxidative stress in the progression of NAFLD (7). Their study found that oxidative stress not only accelerates liver damage but also promotes inflammation through the activation of transcription factors like NFκB, which are crucial in the progression of liver fibrosis (7). This study aligns with our findings, where we observed significantly higher serum PAB levels in individuals with advanced fibrosis (F2 and F3), suggesting that oxidative stress may play a key role in the worsening of liver damage in NAFLD. Our results, combined with those of Franceschetti et al. (7), emphasize the importance of targeting oxidative stress as a potential therapeutic approach for managing NAFLD. Additionally, PAB levels were significantly elevated in the F2 group compared to the non-fibrotic group (F0, F1; *P* < 0.001). Conversely, Nobakht Motlagh Ghoochani et al. did not find significant differences in PAB values between patients with NAFLD and healthy controls (*P* = 0.87), potentially due to study limitations such as small sample size (22). They also noted a positive association between serum PAB levels and BMI (*P* = 0.001) and age (*P* < 0.001) in patients with NAFLD (22). While our results indicate a strong association between PAB levels and fibrosis progression, the underlying mechanisms remain to be fully elucidated. It is plausible that oxidative stress contributes to hepatic stellate cell activation, a key event in fibrogenesis, leading to extracellular matrix deposition and fibrosis progression. Furthermore, the persistence of the association between PAB and fibrosis after adjusting for age, BMI, and comorbidities suggests that oxidative stress may play an independent role in liver disease pathology. However, given that the PAB assay reflects a composite oxidative stress status, further research is needed to determine whether specific oxidative markers within this index are more directly linked to fibrosis progression.

Our study also revealed a notable increase in liver fibrosis severity with higher BMI among people with NAFLD. Consistent with previous findings, Nobakht Motlagh Ghoochani et al. observed significantly higher PAB values in females compared to males in both NAFLD patients and controls (22). There are some other scores developed for this purpose. In this regard, Cho and colleagues found that a higher oxidative balance score, which considers both oxidative stress and antioxidant status, was associated with a reduced risk of developing NAFLD. After adjusting for confounders, participants in the higher quartiles of this score had a significantly lower incidence of NAFLD compared to those in the lowest quartile (23). These findings support the critical role of oxidative stress in NAFLD pathogenesis and emphasize the utility of oxidative stress markers such as the PAB method in assessing disease severity. Further research is needed to explore the clinical applications of PAB and its potential integration into NAFLD management strategies. While the PAB assay provides insights into oxidative stress in NAFLD, several limitations must be considered when evaluating its clinical applicability. The lack of specificity and sensitivity of the PAB assay for oxidative stress biomarkers specific to liver disease raises concerns about potential confounding factors from comorbidities, such as cardiovascular disease or undiagnosed conditions. In addition, the absence of standardization in the assay methodology and reference values across laboratories limits the reproducibility and comparability of results. Moreover, the use of a single-point measurement may not fully reflect the dynamic nature of oxidative stress in chronic diseases like NAFLD, where levels can fluctuate over time.

Oxidative stress promotes hepatocellular injury, inflammation, and extracellular matrix deposition, which are key drivers of fibrosis development (24). Several studies have reported elevated oxidative stress markers in liver diseases such as NAFLD and chronic hepatitis (24), supporting our observation of a positive association between PAB and fibrosis severity. Regarding clinical implications, while our results suggest that PAB may serve as a potential biomarker for fibrosis assessment, its utility remains to be validated. Unlike established non-invasive markers such as the Fibrosis-4 score, PAB has not been widely studied in clinical settings. Further research should assess its diagnostic accuracy compared to existing fibrosis biomarkers and evaluate whether PAB can improve risk stratification in patients with chronic liver disease. Furthermore, interventions targeting oxidative stress—such as antioxidant therapies—should be explored to determine whether modifying PAB levels impacts fibrosis outcomes.

If the results of this study are confirmed in larger studies with the inclusion of multiple factors, and validated against other established tools such as biopsy, we could develop a method to screen patients for this disease, identify those at risk, and initiate timely treatment. This would represent a significant advancement in the management of chronic liver diseases, particularly NAFLD, by enabling early detection and intervention. Such an approach could reduce the burden of advanced fibrosis and its complications, improving patient outcomes and reducing healthcare costs. Our study has a few limitations that should be considered when interpreting the results. First, the study had a limited sample size, which may restrict the generalizability of the findings. Second, we did not account for some potential confounding factors, such as dietary habits, smoking, medication use, and physical activity, which may have influenced the outcomes. Third, the study sample was drawn from a specific region of Iran, and since the prevalence and severity of NAFLD can vary across different ethnic groups and regions, future research should involve a larger, more diverse population to better confirm our findings. Although our results suggest that PAB levels are associated with liver fibrosis in NAFLD, the relatively narrow study population and lack of comprehensive metabolic profiling prevent us from conclusively attributing these findings to NAFLD progression alone. Other metabolic conditions may also contribute to the observed oxidative stress markers, underscoring the need for further research with broader and well-characterized cohorts. In addition, we did not have access to detailed biochemical parameters such as lipid profiles, glucose levels, and inflammatory markers, which could have provided a more comprehensive understanding of the metabolic status of participants. Future studies should use these variables to better elucidate the relationship between oxidative stress, metabolic health, and NAFLD severity.

## Conclusions

Our study highlights the potential of using PAB levels as a predictor for disease progression and fibrosis severity in patients with NAFLD. Furthermore, PAB values were notably higher in NAFLD patients compared to healthy controls, indicating its potential as a potential screening tool for identifying individuals who could benefit from antioxidant therapies. However, given the exploratory nature of our study and the relatively small sample size, larger studies are needed to confirm these results and establish the clinical relevance of PAB in NAFLD management.

## Data Availability

The raw data supporting the conclusions of this article will be made available by the authors, without undue reservation.
